# Loss of SMAD4 Is Associated With Poor Tumor Immunogenicity and Reduced PD-L1 Expression in Pancreatic Cancer

**DOI:** 10.3389/fonc.2022.806963

**Published:** 2022-01-28

**Authors:** Daniel R. Principe, Patrick W. Underwood, Sandeep Kumar, Kaytlin E. Timbers, Regina M. Koch, Jose G. Trevino, Hidayatullah G. Munshi, Ajay Rana

**Affiliations:** ^1^ Medical Scientist Training Program, University of Illinois College of Medicine, Chicago, IL, United States; ^2^ Department of Surgery, University of Illinois at Chicago, Chicago, IL, United States; ^3^ Department of Surgery, University of Florida College of Medicine, Gainesville, FL, United States; ^4^ Department of Surgery, Division of Surgical Oncology, Virginia Commonwealth University, Richmond, VA, United States; ^5^ Department of Medicine, Feinberg School of Medicine, Northwestern University, Chicago, IL, United States; ^6^ Jesse Brown VA Medical Center, Chicago, IL, United States

**Keywords:** pancreatic ductal adenocarcinoma (PDAC), transforming growth factor β (TGFβ), interferon γ (IFNγ), programmed death-ligand 1 (PD-L1), tumor mircorenvironment, tumor immunology

## Abstract

Transforming Growth Factor β (TGFβ) is a key mediator of immune evasion in pancreatic ductal adenocarcinoma (PDAC), and the addition of TGFβ inhibitors in select immunotherapy regimens shows early promise. Though the TGFβ target *SMAD4* is deleted in approximately 55% of PDAC tumors, the effects of SMAD4 loss on tumor immunity have yet to be fully explored. Using a combination of genomic databases and PDAC specimens, we found that tumors with loss of SMAD4 have a comparatively poor T-cell infiltrate. SMAD4 loss was also associated with a reduction in several chemokines with known roles in T-cell recruitment, which was recapitulated using knockdown of *SMAD4* in PDAC cell lines. Accordingly, JURKAT T-cells were poorly attracted to conditioned media from PDAC cells with knockdown of SMAD4 and lost their ability to produce IFNγ. However, while exogenous TGFβ modestly reduced PD-L1 expression in SMAD4-intact cell lines, SMAD4 and PD-L1 positively correlated in human PDAC samples. PD-L1 status was closely related to tumor-infiltrating lymphocytes, particularly IFNγ-producing T-cells, which were more abundant in SMAD4-expressing tumors. Low concentrations of IFNγ upregulated PD-L1 in tumor cells *in vitro*, even when administered alongside high concentrations of TGFβ. Hence, while SMAD4 may have a modest inhibitory effect on PD-L1 in tumor cells, SMAD4 indirectly promotes PD-L1 expression in the pancreatic tumor microenvironment by enhancing T-cell infiltration and IFNγ biosynthesis. These data suggest that pancreatic cancers with loss of *SMAD4* represent a poorly immunogenic disease subtype, and SMAD4 status warrants further exploration as a predictive biomarker for cancer immunotherapy.

## Introduction

Progress in immunotherapy for pancreatic cancer has been difficult. Though immune checkpoint inhibitors (ICIs) have shown therapeutic efficacy in several solid tumors (1–7), clinical trials exploring such approaches in pancreatic cancer have been mostly disappointing, with few showing significant anti-tumor activity (8). There is a notable exception for a particular genomic subgroup of PDAC patients, namely those deficient in DNA mismatch repair. This results in the accumulation of DNA mismatches, which manifest as a microsatellite instability-high (MSI-H) phenotype. The increased mutational burden in MSI-H patients leads to a corresponding increase in the presence of abnormal peptides, many of which are processed and presented as tumor antigens, conferring an increased sensitivity to ICIs (9). Though patients with MSI-H PDAC have derived clinical benefit from ICIs in clinical trials (10), less than 1% of PDAC patients are deficient in mismatch repair, and the majority will not benefit from such an approach (11).

While there is emerging evidence to support additional genomic subtypes of PDAC (12), this has yet to influence either clinical practice or the design of clinical trials exploring immunotherapy in PDAC. Though *KRAS* mutations are ubiquitous in human PDAC tumors, subsequent mutations are highly varied (13). Among the most frequently altered genes in PDAC is *SMAD4*, a tumor suppressor dispensable for normal pancreas development but critical for pancreatic cancer progression (14). *SMAD4*, also known as *deleted in pancreatic carcinoma 4* or *DPC4*, is located on chromosome 18q21 and inactivated in roughly 55% of pancreatic cancers. This occurs either by homozygous deletion (30% of patients) or by intragenic mutations and subsequent loss of heterozygosity (25% of patients) (15). SMAD signaling is a key mediator of the canonical transforming growth factor β (TGFβ) pathway, with important and often contradictory roles in PDAC (16, 17).

In addition to its well-documented effects on tumor cells, TGFβ signaling is emerging as a central mediator of the tumor microenvironment, promoting cancer-associated fibrosis and impeding the effector function of cytotoxic T-lymphocytes (16, 18). Accordingly, TGFβ signaling has been suggested as a key and potentially actionable barrier to the therapeutic efficacy of ICIs in PDAC, particularly when combined with chemotherapy (19, 20). However, though half of PDAC patients will exhibit genetic loss of TGFβ/SMAD signaling in the tumor epithelium, the corresponding alterations in the pancreatic tumor immune microenvironment have yet to be described.

Using a combination of publicly available genomic databases and primary PDAC specimens, we determined that tumors with loss of SMAD4 are poorly immunogenic, with poor T-cell infiltration and a reduction in several chemokines with central roles in T-cell trafficking and effector function. Similarly, conditioned media from PDAC cell lines with ablation of SMAD4 reduced T-cell activation and limited T-cell production of interferon γ (IFNγ) *in vitro*. Additionally, we found that activation of TGFβ/SMAD4 signaling modestly reduced tumor cell expression of PD-L1 *in vitro*; however, SMAD4 and PD-L1 positively correlated in PDAC patient samples.

Notably, we found that PD-L1 status was strongly associated with the degree of tumor-infiltrating lymphocytes, particularly T-cells that stained positive for IFNγ. Low concentrations of IFNγ strongly upregulated PD-L1 in tumor cells *in vitro*, even when administered alongside high concentrations of TGFβ. Hence, while SMAD4 signaling may modestly reduce PD-L1 expression in tumor cells, SMAD4 functions as an indirect inducer of PD-L1 by enhancing the recruitment of tumor-infiltrating IFNγ-producing T-lymphocytes. Overall, these data suggest that pancreatic cancers with loss of *SMAD4* represent a poorly immunogenic molecular subtype, with a relative lack of T-cell infiltration and limited expression of PD-L1. Thus, as immunotherapy advances in the treatment of PDAC, SMAD4-status may warrant consideration as a predictive biomarker for drug responses, particularly those targeting PD-L1/PD-1 signaling.

## Materials & Methods

### Antibodies

All antibodies were purchased from established commercial vendors and were verified by the manufacturer for the specific species and applications for which they were used. A complete list of all antibodies used as well as the vendor, clone, and product numbers can be found in **Table S1**.

### Genomic Database Analysis

As described in our previous studies (20, 21), the provisional TCGA patient dataset (N=186) was downloaded (https://tcgadata.nci.nih.gov/tcga/) and visualized using cBioPortal for Cancer Genomics as described in the original references (22, 23). Genetic analyses were restricted to the 149 fully sequenced tumors, and mRNA values for each gene were determined by comparing RNASeq V2 data in cBioPortal from the rsem.genes.normalized_results file from TCGA microarray data to the gene’s expression distribution in a reference population. All mRNA expression values are plotted in log scale unless otherwise noted, and are displayed with the associated p and Spearmen (S) coefficient values, as well as their respective p values and/or false discovery rate adjusted p vales (q value). For putative copy number alterations, levels of expression are derived from GISTIC/RAE copy-number analysis algorithms and indicate the copy-number level per gene by applying low- and high-level thresholds to the gene copy levels of all the samples.

### Cell Culture

Human pancreatic cancer cells (PANC-1 and MIA PaCa-2) were cultured in DMEM supplemented with 10% heat-inactivated FBS, penicillin (100U/mL), and streptomycin (100mg/ml). AsPC-1 and BxPC-3 cells were grown in RPMI 1640 supplemented with 10% heat-inactivated FBS, penicillin (100U/mL), and streptomycin (100mg/ml). CaPan-1 cells were grown in IMDM1 supplemented with 10% heat-inactivated FBS, penicillin (100U/mL), and streptomycin (100mg/ml), and CaPan-2 cells in McCoy’s 5a Medium also supplemented with 10% heat-inactivated FBS, penicillin (100U/mL), and streptomycin (100mg/ml). Non-malignant HPNE cells were grown in a mixture of modified DMEM and M3 base medium supplemented with 10% heat-inactivated FBS, 10ng/ml human recombinant epidermal growth factor (EGF), penicillin (100U/mL), and streptomycin (100mg/ml). JURKAT cells were maintained in RPMI 1640 medium supplemented with 10% FBS, 1mM L-glutamine, penicillin (100U/mL), and streptomycin (100mg/ml). Cell lines were purchased from the ATCC, used within six months, and kept under passage 10. All cell lines tested negative for mycoplasma *via* PCR within 6 months of use.

### DNA and RNA Transfection

*SMAD4* siRNA (ON-TARGETplus Human SMAD4 siRNA, Catalog ID:L-003902-00-0010, Dharmacon, Lafayette, CO) was reconstituted in nuclease free water per manufacturer specification, delivered at 10nM in RNAiMAX transfection reagent (Invitrogen Waltham, MA), and all knockdowns validated *via* western blotting after 24 hours. The pRK DPC4 Flag (SMAD4^WT^) plasmid has been previously reported (24), and was purchased from Addgene (Watertown, MA, plasmid #12627; http://n2t.net/addgene:12627; RRID: Addgene_12627), expanded in bacterial culture, purified using the Qiagen miniprep kit (Qiagen, Hilden, Germany), and used at a final concentration of 4μg per each well of a 6-well plate.

### qPCR

Quantitative gene expression was performed with gene-specific TaqMan probes, TaqMan Universal PCR Master Mix, and the 7500 Fast Real-time PCR System from Applied Biosystems (Foster City, CA). These data were quantified with the comparative C_T_ method for relative gene expression as described in our previous study (22).

### Western Blot

Cells were lysed in RIPA buffer and homogenized by sonication. Equal amounts of protein (15–50 μg) were mixed with loading dye, boiled for 8 minutes, separated on a denaturing SDS–PAGE gel and transferred to a PVDF membrane. The membrane was blocked in 5% milk/TBS/0.1% Tween for 1 hour and incubated with antibodies against pSMAD2 (Cell Signaling, Danvers, MA, USA), SMAD4, GAPDH (Santa Cruz Biotech, Santa Cruz, CA, USA), PD-L1, or HLA-A, B, C (abcam, Cambridge, MA). The membrane was washed with TBS-0.1% Tween and then incubated with HRP conjugated secondary antibody (Cell Signaling) at room temperature for 1 hour and rewashed. Protein bands were visualized by an enhanced chemiluminescence method (Thermo, Waltham, MA, USA) and resolved digitally per the manufacturer’s specifications.

### Histology, Immunohistochemistry, and Immunofluorescence

Tissues were fixed in 10% formalin, paraffin-embedded, and sections at 4μm interval were cut from each tissue, and stained with hematoxylin and eosin (H&E), or *via* immunohistochemistry (IHC) or immunofluorescence (IF). For immunohistochemistry, slides were deparaffinized by xylenes and rehydrated by ethanol gradient, then heated in a pressure cooker using DAKO retrieval buffer (DAKO, Santa Clara, CA). Endogenous peroxidases were quenched in 3% hydrogen peroxide in methanol for 30 minutes. Tissues were blocked with 0.5% BSA in PBS for 30 minutes and incubated with primary antibodies against: SMAD4, CD3 (Santa Cruz), CD45, or PD-L1 (Cell Signaling) at 1:50–1:200 overnight at 4°C. Slides were developed using HRP-conjugated secondary antibodies followed by DAB substrate/buffer (DAKO).

For immunofluorescence, slides were heated *via* pressure cooker in DAKO retrieval buffer and tissues blocked with 0.5% BSA in PBS for 1 hour at room temperature. Sections were exposed to primary antibodies against CK19 (University of Iowa Hybridoma Bank), E-Cadherin (Cell Signaling), CD3, (Santa Cruz), or IFNγ (abcam) at 1:50–1:200 overnight at 4°C. Slides were developed using AlexaFluor 488- or 594-conjugated secondary antibodies (1:200–1:1,000, abcam), mounted in DAPI-containing media (Santa Cruz Biotechnology), exposed to DAPI, FITC, and Texas Red filters.

### Microscopy

All images were acquired using a Nikon 40x-400x Epi-Fluorescent Inverted Microscope with Phase Contrast Kit and Nikon bright-field camera attachment. Negative slides were used for white balance, and for all images no analog or digital gain was used. For fluorescent imaging, we used positive control slides for each experiment and auto-exposed slides using Nikon NIS elements software using a gain setting of zero. Gain was similarly set to zero and LUTs were used to reduce background based on negative control slides. These LUT values and exposure times were standardized and used for all other similarly stained slides. Images were superimposed also using Nikon NIS elements software.

### Tissue Slide Counts, Scores, and Measurements

All counts were performed by a minimum of three blinded investigators and each value displayed includes the average of minimum of three high power fields per specimen. All counts from each investigator were averaged and value distributions were visualized *via* Minitab express software, showing the median value as a solid line, as well as each quartile of all additional values excluding any statistical outliers.

### Flow Cytometry

JURKAT cells were washed in PBS, incubated with a Golgi plug/protein transport inhibitor (BD biosciences, San Jose, CA), and stained with CD69-APC (BioLegend, San Diego, CA) and an Alive/Dead kit (Invitrogen, Grand Island, NY) at 1:200-1:1000 in PBS at room temperature for 40 minutes. Cells were then fixed with 1% PFA in PBS for 10 minutes at room temperature, and select groups stained with anti-IFNγ-PE (BioLegend, San Diego, CA) at 1:100 in perm/stain buffer (BD biosciences) for 30 minutes over ice and washed three times with perm/wash buffer (BD biosciences). Cells were analyzed with a BD Fortessa Cytometer, gating exclusively to cells within acceptable FSC/SSC parameters. All subsequent flow plots correspond to live, single cells based on Live/Dead assay and SSC-W gating, and are representative of 100,000 events unless otherwise stated. High and low populations were identified based on the geometric mean of the control group, based on unstained and isotype controls for each antibody. All other experiments were compared to both unstained, single cell, and isotype controls.

### Study Approval

All experiments involving the use of human specimens were performed using a PDAC tumor microarray described previously (18, 19), with tissues were obtained in a de-identified manner from patients who provided fully informed consent and following local IRB approval at Northwestern University, or from the University of Florida, also following local IRB approval and from fully consenting patients.

### Statistical Analysis

Data were analyzed by either Student’s T-test, hazard ratio test, simple linear regression analysis, or ANOVA fit to a general linear model in Minitab express, the validity of which was tested by adherence to the normality assumption and the fitted plot of the residuals. Results were arranged by the Tukey method, and considered significant at p < 0.05 unless otherwise noted. Results are presented as either boxplot showing the median value and all other values arranged into quartiles, individual value plot showing the median value, or as the mean of individual replicates plus standard deviation.

## Results

### Tumors With Loss of SMAD4 Display Reduced Lymphocyte Infiltration Independent of Neoadjuvant Chemotherapy Status

To identify potential immunologic differences between SMAD4-expressing and SMAD4-nonexpressing tumors, we first evaluated the TCGA genomic database of pancreatic cancer patients (N=186). For the 149 patients with fully sequenced tumors, 71 (47.7%) had a presumptive loss of *SMAD4*, either through an inactivating genetic mutation (14.8%), copy number deletion (9.4%), mRNA downregulation (2.0%), or more than one of these alterations (21.5%) (**Figures S1A, B**). Consistent with previous reports (23), patients with any *SMAD4* alteration had poorer progression-free survival (PFS) when compared to those without *SMAD4* alteration (**Figure S1C**).

We next evaluated the comparative expression of several immune-associated genes within these groups and determined that patients with wild type *SMAD4* had a highly significant increase in mRNA expression of the pan T-cell markers *CD3E* and *CD3G* (**Figures S1D, E**). Similarly, *SMAD4* wild-type patients also had increased mRNA expression of the cytotoxic T-cell surrogates CD8A and CD8B (**Figures S1F, G**). *SMAD4* was strongly associated with mRNA expression of GranzymeB (*GZMB*) and Perforin (*PRF1*), two functional markers of T-cell mediated cytotoxicity (**Figures S1H, I**). We also determined the relationship between *SMAD4* mRNA expression and *CD3E*, *CD8A*, or *PRF1* mRNA and found that each has a significant positive association with SMAD4 (**Figures S1J-L**). We observed similar results regarding mRNA expression of the Type 2 TGFβ Receptor (*TGFBR2)*, which was also positively associated with several T-cell surrogates and markers of T-cell mediated cytotoxicity (**Figures S2A-F**).

Additionally, *SMAD4* mRNA expression was strongly associated with that of the natural killer cell marker *NCAM1* (CD56) (**Figure S3A**). We did not find a significant association between *SMAD4* expression and that of the macrophage surrogate *CD68* (**Figure S3B**), only a modest association with the dendritic cell marker *ITGAX* (CD11c) (**Figure S3C**), and no association with the neutrophil-associated marker *CEACAM8* (CD66b) (**Figure S3D**). However, *SMAD4* expression was positively associated with B-cell markers *CD19* and *MS4A1* (CD20) (**Figures S3E, F**).

**Figure 1:**
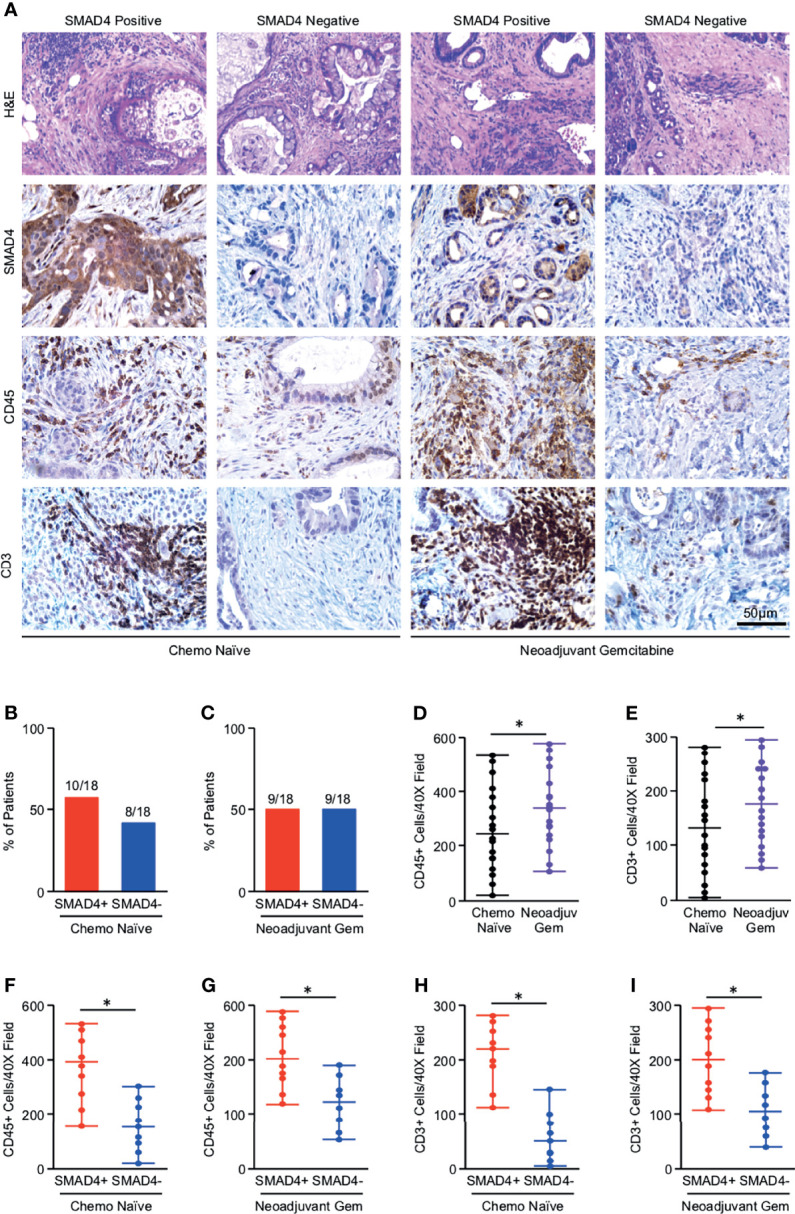
Tumors with loss of SMAD4 display reduced lymphocyte infiltration independent of neoadjuvant chemotherapy status. **(A)** Excisional biopsies from 36 PDAC patients were sectioned and stained either with H&E or *via* immunohistochemistry for SMAD4, the pan-leukocyte antigen CD45, or T-cell marker CD3 and representative images shown for each from either chemotherapy naïve patients (N=18) or patients who had received neoadjuvant Gemcitabine-based chemotherapy (N=18). **(B, C)** The percent of patients from either the chemo-naïve or neoadjuvant Gemcitabine group that was either SMAD4-expressing (SMAD4+) or SMAD4-non-expressing (SMAD4-). **(D, E)** The number of CD45+ or CD3+ cells per 40X field was quantified by three blinded investigators, related to chemotherapy status, and displayed as an individual value plot. Using these values, the number of CD45 positive cells was next related to SMAD4 status in either **(F)** the chemo naïve group or **(G)** the neoadjuvant Gemcitabine group and displayed as an individual value plot. **(H, I)** The number of CD3+ T-cells were quantified as described and related to SMAD4 status in either the chemo-naïve group or the neoadjuvant Gemcitabine group and displayed as an individual value plot. (*p < 0.05).

Given the limitations of using bulk tumor mRNA sequencing data from publicly available datasets, we next explored the relationship between SMAD4 expression and tumor immunogenicity in 36 human PDAC excisional biopsies. Eighteen of these patients were chemotherapy naïve at the time of surgery, and 18 had received neoadjuvant Gemcitabine-based chemotherapy. Clinical characteristics describing this patient cohort are shown in **Table S2**. Tissues were sectioned and stained either with H&E or by immunohistochemistry for SMAD4, the pan-leukocyte marker CD45, or the T-cell lineage marker CD3 (**Figure 1A**). By IHC, SMAD4 was detected in 10/18 (44%) of chemotherapy-naïve PDAC tumors and 9/18 (50%) of chemotherapy-treated PDAC tumors (**Figures 1B, C**). CD45+ tumor-infiltrating leukocytes were highly varied across all tumor specimens but were slightly elevated in tumors treated with neoadjuvant chemotherapy, as were cells that stained positive for CD3 (**Figures 1D, E**). Much like those in the TCGA dataset, SMAD4-expressing human PDAC tumors had a higher median expression of CD45+ cells independent of neoadjuvant chemotherapy (**Figures 1F, G**), with similar results observed with CD3+ T-cells (**Figures 1H, I**).

### SMAD4 Expression and Increased T-Cell Infiltration Predict for Better Overall Survival in PDAC Patients

**Figure 2:**
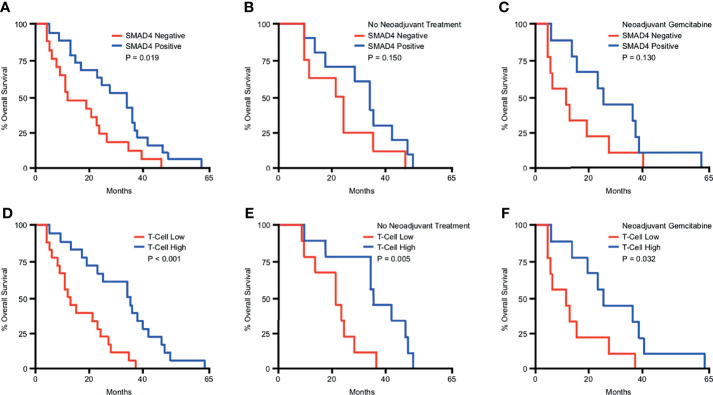
SMAD4 expression and increased T-cell infiltration predict for improved overall survival in PDAC patients. Kaplan-Meier curve indicating months of overall survival following surgical resection for a cohort of 36 patients arranged by: **(A)** SMAD4 status determined by immunohistochemistry, **(B)** SMAD4 status for only the patients that received no neoadjuvant therapy, **(C)** SMAD4 status for only the patients that received neoadjuvant Gemcitabine-based chemotherapy, **(D)** Patients above or below the median value for CD3+ T-cells per high power field, **(E)** T-cells status for only the patients that received no neoadjuvant therapy, **(F)** T-cells status for only the patients that received neoadjuvant Gemcitabine-based chemotherapy.

To determine the prognostic significance of these observations, we next evaluated the relationship between SMAD4 status, T-cell infiltration, and overall survival in our patient cohort. In this group of patients, the median overall survival after surgery was 22.85 months. Those with intact SMAD4 expression demonstrated a significant survival advantage compared to those with presumptive loss of SMAD4 (**Figure 2A**). However, this did not achieve statistical significance when separating patients by treatment status, most likely due to the reduced sample size in each group (**Figures 2B, C**). Patients with high (above the median value) T-cell infiltration demonstrated a highly significant survival advantage, which was statistically significant even when grouping patients by treatment status (**Figures 2D–F**).

Overall survival was not significantly affected by natal sex, the type of surgery administered, the size of the primary tumor, nor tumor differentiation on pathologic evaluation (**Figure S4A-D**). Survival was significantly affected by the degree of lymph node involvement, as patients with node-negative disease had significantly improved overall survival compared to those with lymph node involvement (**Figure S4E**). Outcomes did not significantly differ between patients that did and did not receive neoadjuvant treatment (**Figure S4F**).

### Loss of SMAD4 Impairs CCL, CXCL, and IL-Family Cytokine Synthesis in PDAC Cells

Given the apparent alteration in the immunogenicity of SMAD4-expressing and non-expressing tumors, we next revisited the TCGA cohort of PDAC patient mRNA samples and explored the relationship between expression of *SMAD4* and that of the known CCL/CXCL family chemokines and interleukin family cytokines. Consistent with an overall increase in tumor immunogenicity, *SMAD4* mRNA had significant (FRD adjusted p value > 0.05) positive associations with *CCL2, 4, 5, 8, 11, 14, 16, 17, 19, 21, 22, 23*, and *25* (**Table S3**). *SMAD4* was less frequently correlated with CXCL-family members, with significant positive associations with *CXCL9, 12*, and *13*, and significant inverse associations with *CXCL16* and *17* (**Table S3**). The relationship between *SMAD4* and interleukin family cytokines was more varied, with significant positive associations between *SMAD4* and *IL2, 6, 10, 12A, 12B, 13, 15, 16, 17F, 17D, 24, 26, 33*, and *34*. SMAD4 had significant negative associations with I*L1A, 17C, 18, 36G*, and *36B* (**Table S3**).

**Figure 3:**
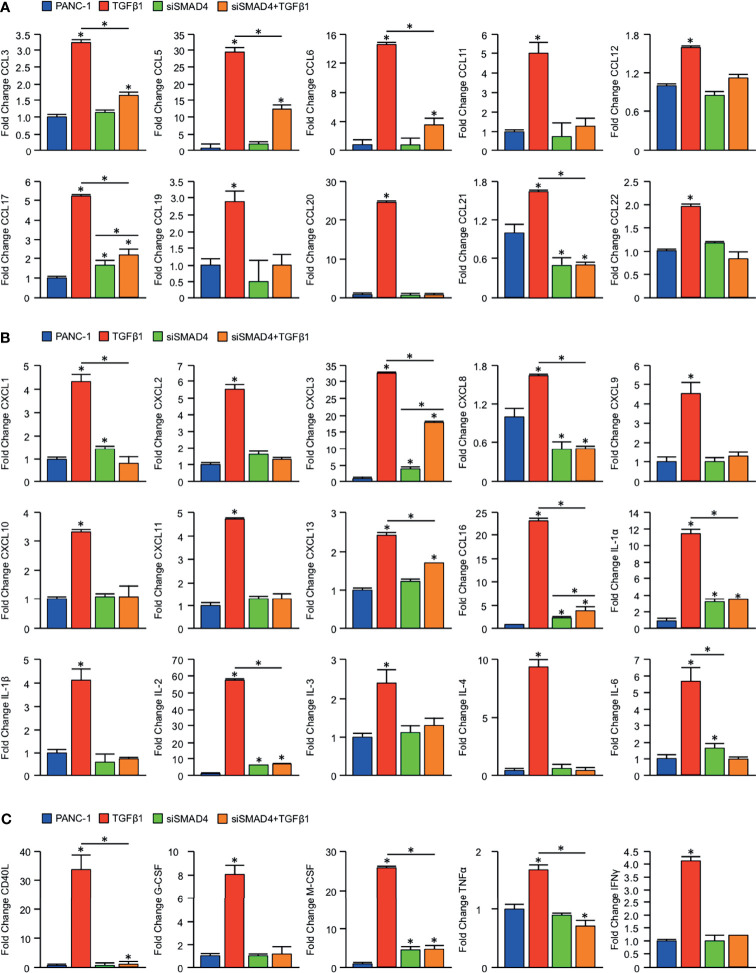
Loss of SMAD4 impairs CCL, CXCL, and IL-family cytokine synthesis in PDAC cells. An equal number of PANC-1 cells were seeded into 6-well plates and incubated either with a control siRNA (siControl) or siRNA against SMAD4 (siSMAD4). After 24 hours, cells were treated with either a saline vehicle or 10ng/mL of recombinant TGFβ1. Following another 24 hours, cells were incubated with a protein transport inhibitor for one hour, lysed, and 200μg of total cell lysate was evaluated by a high throughput proteome profiler array (ARY022B). Pixel density was evaluated using ImageJ, and samples normalized to the mean intensity of the reference spots for each blot minus the background density. Values are presented as fold change for **(A)** CCL family cytokines/chemokines, **(B)** CXCL and IL family cytokines/chemokines, **(C)** additional immunomodulatory proteins. (*p < 0.05).

To determine whether loss of SMAD4 has a direct effect on tumor cytokine synthesis, we first used the established SMAD4-intact PDAC cell line PANC-1. PANC-1 cells were incubated with either a control siRNA (siControl) or siRNA directed against SMAD4 (siSMAD4). After 24 hours, cells were treated with either a saline vehicle or 10ng/mL of recombinant TGFβ1. Following another 24 hours, cells were lysed and subjected to a high throughput array of 105 human immunoregulatory proteins (**Figure S5A**). After normalizing to reference samples, we identified consistent alterations to the secretome of tumor cells treated with TGFβ1, with highly significant increases in the expression of CCL3, 5, 6, 11, 12, 17, 19, 20, 21, and 22. However, this was not observed in siSMAD4 cells treated with TGFβ1, which only displayed a modest increase in CCL17 and 21 (**Figure 3A**).

We observed similar results regarding CXCL and IL family members, with TGFβ1 leading to substantial increases in CXCL1, 2, 3, 8, 9, 10, 11, 13, and 16, as well as IL-1α, 1β, 2, 3, 4, and 6. Again, this was not observed in siSMAD4 cells treated with TGFβ1, where we found only modest increases in CXCL3, 8, 13, 16, IL-1α, and IL-2 (**Figure 3B**). Beyond the effects on CCL and CXCL family chemokines, TGFβ1 also led to significant SMAD-dependent alterations to additional immunomodulators, including a nearly 30-fold increase in the co-stimulatory surface protein CD40L, as well as similarly significant increases in inflammatory cytokines such as G-CSF, M-CSF, TNFα, and IFNγ (**Figure 3C**).

We repeated this experiment using the poorly TGFβ-responsive cell line MIA PaCa-2, which has low expression of TGFBR2 and is refractory from TGFβ-induced cell cycle arrest (25, 26). However, these cells express other TGFβ receptors, and recent reports suggest that they are at least partly TGFβ-responsive, undergoing SMAD2 phosphorylation on TGFβ stimulation (25, 26). In our hands these cells indeed have low, but not zero, TGFBR2 expression (**Figure S5B**). Accordingly, exogenous TGFβ had a more modest effect on cytokine profiling, though the few cytokines induced by TGFβ were mitigated in cells with SMAD4 knockdown (**Figure S5C**).

To determine whether this corresponds to an increase in T-cell chemotaxis, we conducted migration assays using immortalized JURKAT T-cells and PANC-1-conditioned media. JURKAT cells were starved of growth supplements overnight, seeded in transwell chambers in serum-free media, and introduced to conditioned media from either PANC-1 cells treated with either siControl or siSMAD4, both with and without 10ng/mL TGFβ1. We found that JURKAT cells were modestly attracted to media conditioned with siControl-treated PANC-1 cells for 24 hours compared to control media. JURKAT cells were strongly attracted to the conditioned media of PANC-1 cells stimulated with 10ng/mL TGFβ1. Contrastingly, JURKAT cells were poorly attracted to the conditioned media of PANC-1 administered siSMAD4, which was not significantly enhanced by the addition of TGFβ1 (**Figure S5D**).

### JURKAT T-Cells Remain Refractory From Full Activation When Grown in Conditioned Media From SMAD4-Deficient Tumor Cells

To determine whether the observed SMAD4-dependent alterations in cytokine and chemokine production correspond to changes in T-cell activation, we again utilized PANC-1 tumor cells, which were incubated with either a control siRNA (siControl) or siRNA against *SMAD4* (siSMAD4). After 24 hours, cells were stimulated with 10ng/mL of recombinant TGFβ1, and media was changed to serum-free DMEM four hours later. These media were collected after another 24 hours, supplemented with 10% FBS and 2μl/mL ImmunoCult Human CD3/CD28 T-Cell Activator, and administered to 1 million serum-starved JURKAT T-cells. After 24 hours, these JURKAT cells were analyzed for T-cell activation by flow cytometry for the activation markers CD69 and IFNγ.

**Figure 4:**
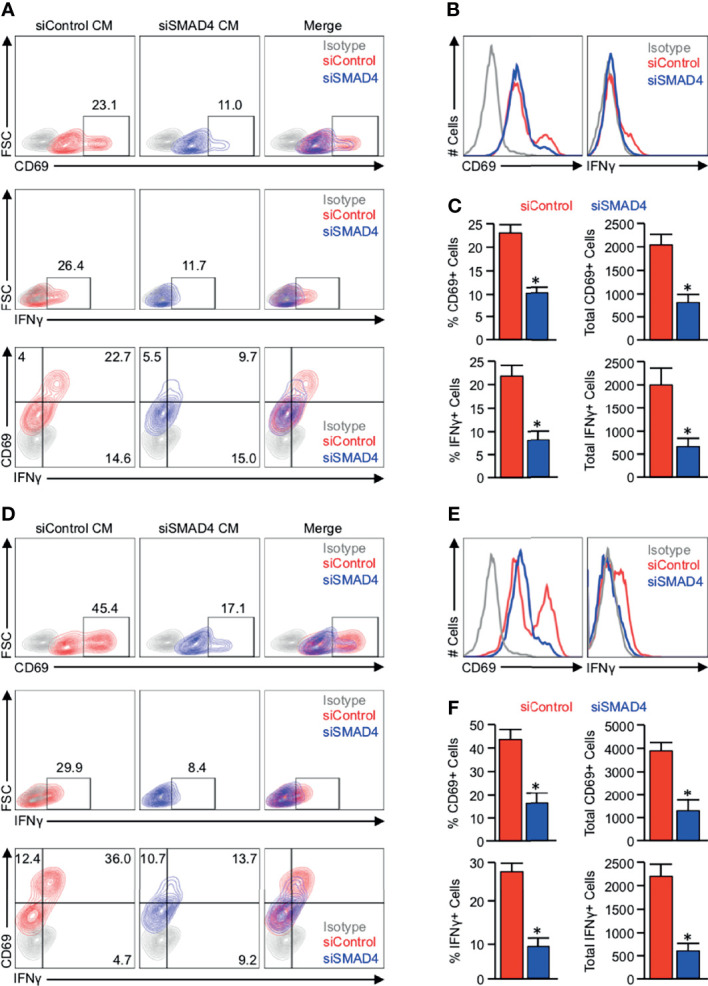
JURKAT T-cells remain refractory from full activation when grown in conditioned media from SMAD4-deficient tumor cells. **(A)** PANC-1 tumor cells were incubated with either a control siRNA (siControl) or siRNA against *SMAD4* (siSMAD4) and stimulated with 10ng/mL of recombinant TGFβ1 after 24 hours. Four hours after treatment, media was changed to serum-free DMEM and collected after another 24 hours. This media was supplemented with 10% FBS and 2μl/mL ImmunoCult Human CD3/CD28 T Cell Activator and administered to 1 million serum-starved JURKAT T-cells. After 24 hours, JURKAT cells were collected, incubated with a protein transport inhibitor for one hour, and analyzed for T-cell activation by flow cytometry for the activation markers CD69, IFNγ, or CD69 and IFNγ. **(B)** The modal expression of CD69 and IFNγ is displayed as a histogram plot. **(C)** Using the described gating, the relative percent of CD69+ and IFNγ+ events are plotted, as are the absolute number of each per 10,000 events (*p < 0.05). **(D)** MIA PaCa-2 tumor cells were incubated with either a siControl or siSMAD4, treated similarly, and media collected as described. This media was supplemented with 10% FBS and 2μl/mL ImmunoCult Human CD3/CD28 T Cell Activator, and administered to 1 million serum-starved JURKAT T-cells, which were analyzed by flow cytometry as described previously. **(E)** The modal expression of CD69 and IFNγ is displayed as a histogram plot. **(F)** Using the described gating, the relative percent of CD69+ and IFNγ+ events are plotted, as are the absolute number of each per 10,000 events. (*p < 0.05).

While JURKAT T-cells were able to remain predominantly active in the presence of conditioned media from PANC-1 cells treated with siControl, we observed a substantial reduction in CD69+ and IFNγ+ expressing cells for those grown in conditioned media from PANC-1 cells treated with siSMAD4 (**Figures 4A–C**, **S6**). We repeated these experiments using conditioned media from similarly treated MIA PaCa-2 cells. We again found that JURKAT cells incubated in conditioned media from MIA PaCa-2 cells administered siControl maintained robust CD69 and IFNγ expression after 24 hours, which was reduced in JURKAT cells grown in conditioned media from MIA PaCa-2 treated with siSMAD4 (**Figures 4D–F**).

### TGFβ/SMAD Signaling Downregulates PD-L1 Expression *In Vitro*, Yet SMAD4-Intact Tumors Have Higher Expression of PD-L1 *In Vivo*

**Figure 5:**
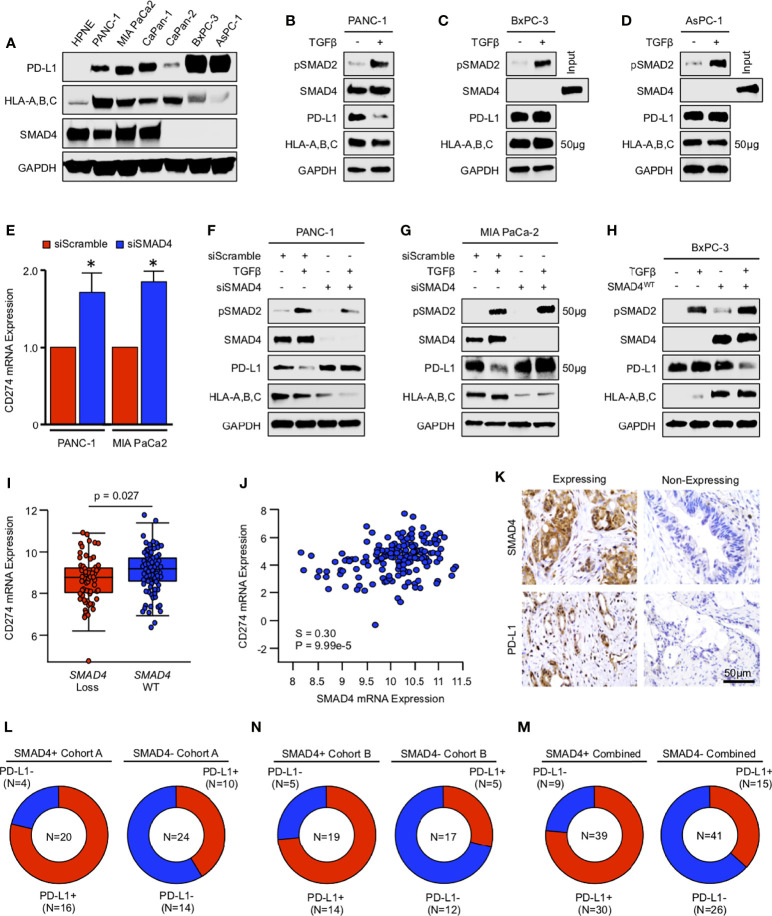
TGFβ/SMAD signaling downregulates PD-L1 expression *in vitro*, yet SMAD4-intact tumors have higher expression of PD-L1 *in vivo.* **(A)** Non-malignant HPNE cells and human PDAC cell lines PANC-1, MIA PaCa-2, CaPan-1, CaPan-2, BxPC-3, and AsPC-1 were lysed and analyzed for basal expression of SMAD4, PD-L1, and HLA-A,B,C by western blot. **(B-D)** PANC-1, BxPC-3, and AsPC-1 cells were incubated with either a saline vehicle or 10ng/mL of recombinant TGFβ1 and evaluated after 24 hours by western blot. **(E)** PANC-1 and MIA PaCa-2 cells were incubated with either a control siRNA (siControl) or siRNA against *SMAD4* (siSMAD4), and after 24 hours, *CD274* (PD-L1) mRNA was evaluated by qPCR. **(F, G)** PANC-1 and MIA PaCa-2 cells were incubated with either siControl or siSMAD4 and, after 24 hours, stimulated with 10ng/mL of recombinant TGFβ1. Cells were lysed after another 24-hour period and evaluated by western blot analysis. **(H)** BxPC-3 cells were transfected with a wild-type SMAD4 plasmid (SMAD4^WT^). After 24 hours, cells were stimulated with 10ng/mL of recombinant TGFβ1 and evaluated by western blot analysis after another 24 hours. **(I)** Using the TCGA genomic database of pancreatic cancer patients (N=186), the 149 fully sequenced tumors were separated into two groups: those with no SMAD4 alteration (SMAD4 wild-type or WT), and those with presumptive SMAD4 loss *via* a known inactivating mutation, mRNA downregulation, and/or copy number deletion. We then compared the mRNA expression of *CD274* in each group. **(J)** *SMAD4* mRNA expression was plotted against that of *CD274*. All mRNA expression values are plotted in log scale and are displayed with the associated p and Spearmen (S) coefficient values. **(K)** Excisional biopsies from two cohorts of PDAC patients (N=44 and N=36, respectively) were sectioned and stained *via* immunohistochemistry for SMAD4 or PD-L1. **(L-N)** Patients were grouped as being either SMAD4-expressing (SMAD4+) or SMAD4-non-expressing (SMAD4-), and the percent of each group also positive for PD-L1 displayed as a pie chart. (*p < 0.05).

To determine the impact of SMAD4 loss on additional immune cell processes, particularly those related to cancer immunotherapy, we next explored the relationship between SMAD4 and PD-L1 *in vitro*, first evaluating the basal expression of PD-L1 in a variety of established PDAC cell lines. While all tumor cell lines had increased PD-L1 expression compared to non-malignant HPNE cells, PD-L1 expression was highest in BxPC-3 cells with homozygous deletion of *SMAD4* and AsPC-1 cells harboring an inactivating *SMAD4* mutation (**Figure 5A**). Interestingly, despite reduced levels of functionally intact SMAD4, CaPan-2 cells had relatively low levels of PD-L1 (**Figure 5A**). We observed similar results regarding HLA-A,B,C, with particularly low levels of expression in BxPC-3 and AsPC-1 cells (**Figure 5A**).

Using PANC-1 cells as a model of SMAD4-intact PDAC and both BxPC-3 and AsPC-1 cells as a model of SMAD4-deficient PDAC, we next incubated tumor cells with 10ng/mL recombinant TGFβ1 for 24 hours and evaluated the expression of PD-L1 by western blot. In PANC-1 cells, TGFβ led to significant downregulation of PD-L1 (**Figure 5B**), though TGFβ did not affect PD-L1 expression in either BxPC-3 or AsPC-1 cells (**Figures 5C, D**). We next used the SMAD4-expressing PANC-1 and MIA PaCa-2 PDAC cell lines and administered either siControl or siSMAD4. After 24 hours, *CD274* mRNA was evaluated by qPCR. In both cell lines, we observed a significant reduction in *CD274* mRNA in both PANC-1 and MIA PaCa-2 cells 24 hours after administration of siSMAD4 (**Figure 5E**).

To determine whether TGFβ-induced suppression of PD-L1 is indeed SMAD4-dependent, we next administered PANC-1 and MIA PaCa-2 cells either siControl or siSMAD4 as described. After 24 hours, cells were then incubated with 10ng/mL TGFβ1 and evaluated by western blot after an additional 24-hour period. As previously, exogenous TGFβ1 led to the repression of PD-L1 expression in PANC-1 cells incubated with the siControl control, yet failed to repress PD-L1 in cells incubated with siSMAD4 (**Figure 5F**). Despite the limited TGFBR2 expression in the in MIA PaCa-2 cell line, high dose TGFβ1 still modestly reduced PD-L1 expression by western blot, which was not observed in cells with siSMAD4 (**Figure 5G**). We next performed a similar experiment restoring SMAD4 expression in BxPC-3 cells using a wild-type SMAD4 plasmid (SMAD4^WT^). Cells were transfected and, after 24 hours, incubated with 10ng/mL TGFβ1. Cells were lysed after another 24 hours, and PD-L1 expression was analyzed by western blot. As previously, exogenous TGFβ1 did not affect PD-L1 expression in BXPC-3 cells. However, following the restoration of SMAD4 expression, exogenous TGFβ1 effectively repressed PD-L1 (**Figure 5H**).

We next explored the relationship between SMAD4 and PD-L1 expression *in vivo*, first using the TCGA cohort as described previously. Interestingly, we observed a comparative increase in *CD274* mRNA expression in patients with SMAD4-intact tumors compared to those with presumptive loss of SMAD4 (**Figure 5I**), paralleled by a highly significant, positive association between *SMAD4* and *CD274* mRNA expression (**Figure 5J**). Using the aforementioned cohort of 36 excisional biopsies, we stained tissues for PD-L1 and related this to SMAD4 status (**Figure 5K**), as well as analyzed the relationship between these two proteins using a separate cohort of 44 archived specimens with 14 adjacent non-malignant tissues that had previously been stained for SMAD4 (18) and PD-L1 (19). In these tissues, PD-L1 was expressed in 2/14 (14.3%) adjacent non-malignant tissues, 26/44 (59.1%) archived PDAC tissues (Cohort A), and 19/36 (52.8%) specimens from the most recent group of 36 patients (Cohort B). When PDAC cohorts were combined, PD-L1 was expressed in 45/80 (56.3%) PDAC tissues. SMAD4 was expressed in 13/14 (92.9%) adjacent non-malignant samples, 20/44 (45.5%) PDAC samples in cohort A, 19/36 (52.8%) in cohort B, and 39/80 (48.8%) in the combined PDAC cohort.

Of the 20 patients in cohort A that expressed SMAD4, 16 (80%) also expressed PD-L1, whereas only 4 (20%) did not. Conversely, of the 24 patients without detectable SMAD4 expression, only 10 (41.7%) expressed PD-L1 (**Figure 5L**). We observed similar results in cohort B, where 14/19 (73.7%) patients with expression of SMAD4 also had expression of PD-L1, compared to only 5/17 (29.4%) patients in the SMAD4-negative group (**Figure 5M**). Combined, 30 of 39 (76.9%) of SMAD4-expressing patients were positive for PD-L1, compared to 15 of 41 (36.6%) of SMAD4-nonexpressing patients (**Figure 5N**).

### IFNγ Overcomes the Inhibitory Effects of TGFβ/SMAD Signaling on PD-L1 expression Both *In Vivo* and *In Vitro*

**Figure 6:**
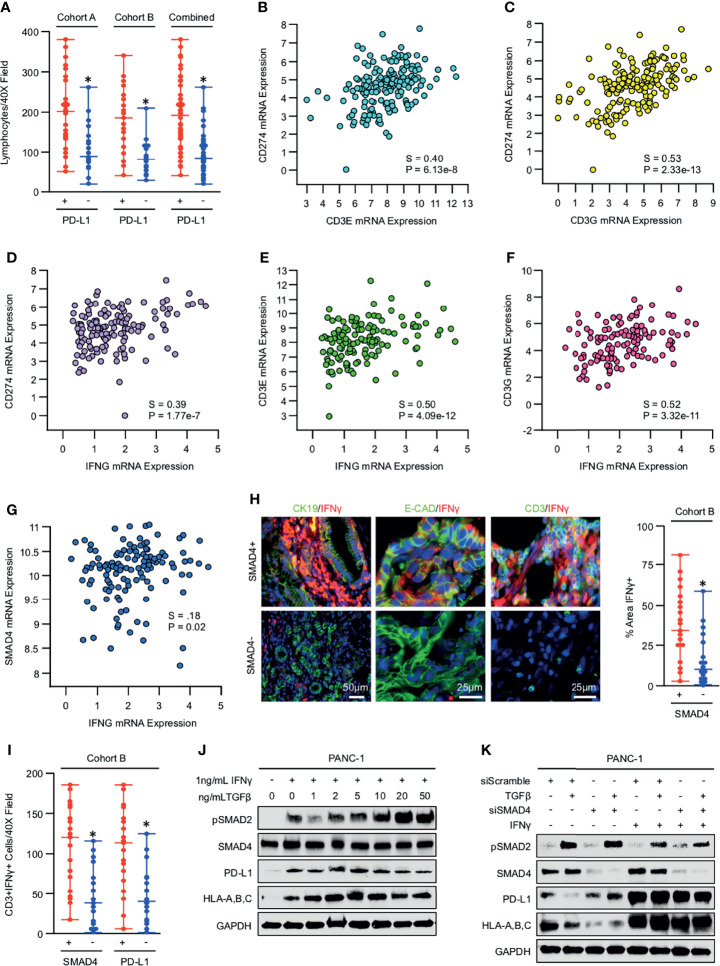
IFNγ overcomes the inhibitory effects of TGFβ/SMAD signaling on PD-L1 expression both *in vivo* and *in vitro.* **(A)** Excisional biopsies from 44 PDAC patients in cohort A and 36 PDAC patients in cohort B were sectioned and stained with H&E, lymphocytes quantified per 40X field, and arranged by PD-L1 status. **(B-D)** Using the TCGA genomic database of pancreatic cancer patients, *CD274* mRNA expression was plotted against that of *CD3E*, *CD3G*, and *IFNG*. **(E-G)** Also, using the TCGA genomic database, *IFNG* mRNA expression was plotted against that of *CD3E*, *CD3G*, or *SMAD4.* ***(*H*)*** The 36 excisional PDAC specimens from cohort B were stained by immunohistochemistry for IFNγ, as well as dual-stained for either the duct marker CK19 and IFNγ, epithelial surrogate marker E-Cadherin and IFNγ, or the T-cell marker CD3 and IFNγ. The percent area positive for IFNγ was quantified as described and related to SMAD4 status. **(I)** The number of CD3+IFNγ+ cells were quantified per 40X field and arranged by both SMAD4 and PD-L1 status. **(J)** PANC-1 cells were incubated with 1ng/mL of recombinant IFNγ in the presence of increasing doses of recombinant TGFβ1, and PD-L1 expression evaluated by western blot after 24 hours. **(K)** PANC-1 cells were again incubated with either siControl or siSMAD4 and, after 24 hours, stimulated with 10ng/mL of recombinant TGFβ1. This experiment was also conducted in the presence of 1ng/mL recombinant IFNγ given concurrently with TGFβ1, and 24-hours after stimulation, cells were evaluated by western blot. (*p < 0.05).

Though our *in vitro* data appear to suggest that SMAD signals impede PD-L1 expression, SMAD4 and PD-L1 expression are positively associated in human PDAC specimens. Given that the relationship between SMAD4 was highly predictive for both PD-L1 expression and increased lymphocyte infiltration, we also evaluated the association between PD-L1 and tumor-infiltrating lymphocytes. In both patient cohorts, tissues that stained positive for PD-L1 had a comparative increase in tumor-infiltrating lymphocytes (**Figure 6A**). We found a similar relationship using the TCGA dataset, where *CD274* positively correlated with mRNA expression of T-cell surrogate markers *CD3E* and *CD3G* (**Figures 6B, C**), as well as that of the IFNγ gene *IFNG* (**Figure 6D**). Expression of both *CD3E* and *CD3G* was closely related to that of *IFNG* (**Figures 6E, F**), and consistent with our previous data, *SMAD4* expression also was positively correlated with that of *IFNG* (**Figure 6G**).

We next stained the 36 tumor specimens for IFNγ by immunohistochemistry and found that IFNγ expression localized predominantly to the CD3+ T-cell infiltrate, with some staining also co-localizing CK19 and E-Cadherin-expressing epithelial tissues (**Figure 6H**). Consistent with TCGA data, tumor specimens with SMAD4 expression displayed a significant increase in the percent area that stained positive for IFNγ (**Figure 6H**), and tumors that expressed SMAD4 or PD-L1 demonstrated a highly significant increase in the degree of tumor-infiltrating T-cells that also stained positive IFNγ (**Figure 6I**).

As IFNγ is a well-established inducer of PD-L1 (27), we next sought to determine whether the increased levels of IFNγ observed in SMAD4-expressing tumors was enough to overcome the suppressive effects of TGFβ/SMAD signals on PD-L1 expression *in vitro*. PANC-1 cells were first incubated with 1ng/mL of recombinant IFNγ, as well as increasing concentrations of TGFβ1 and PD-L1 levels evaluated by western blot after 24 hours. As expected, IFNγ strongly enhanced PD-L1 expression, though this was not affected by the addition of up to 50ng/mL TGFβ1 (**Figure 6J**). We repeated our previous experiment using PANC-1 cells incubated with siControl or siSMAD4 and found that again TGFβ1 modestly reduced PD-L1 expression in a SMAD4-dependent manner in the absence of low-dose IFNγ but did not affect PD-L1 expression in the presence of 1ng/mL IFNγ, where all groups showed strong upregulation of PD-L1 (**Figure 6K**).

## Discussion

Pancreatic cancer is associated with poor clinical outcomes, in part attributed to limited therapeutic responses to standard treatment regimens (28). While recent data has suggested that there are distinct genomic subsets of PDAC tumors (29), molecular profiling has yet to substantially impact treatment decisions for PDAC with the exception of PARP inhibition for tumors with loss of high-fidelity double-strand break homologous recombination (21, 30) or the anti-PD-1 antibody Pembrolizumab for those deficient in DNA mismatch repair (dMMR) with high microsatellite instability (MSI-H) (10). As these criteria will apply to very few patients, nearly all PDAC patients are treated similarly with a combination of surgery, if possible, and aggressive chemotherapy (28). Hence, there is a need to identify additional molecular subtypes of PDAC in hopes of matching these tumors to a more effective treatment strategy. In that regard, the mutational landscape of PDAC is highly varied (29). Though oncogenic KRAS mutations are ubiquitous in PDAC (31), nearly half of patients harbor genetic inactivation of the tumor suppressor gene *SMAD4* (23). We have previously identified TGFβ, the upstream activator of SMAD4, as a potential immune checkpoint in PDAC (18–20). Accordingly, TGFβ pathway inhibition is showing early promise in clinical trial for PDAC patients, particularly when combined with chemo- (32) or immunotherapy (33). While related trials are ongoing (34), to our knowledge, no studies have examined whether patients with genetic defects in the TGFβ/SMAD4 signaling pathway will have alterations in local immune function.

Using a combination of publicly available genomic databases and excisional biopsies, we determined that the loss of *SMAD4* is associated with the impaired recruitment of a variety of leukocyte subsets, most notably cytotoxic T-cells. As these cells are central to the efficacy of ICIs, which are currently under investigation in PDAC, we subsequently examined the relationship between SMAD4 and clinically actionable immune checkpoints. While SMAD4 was strongly associated with PD-L1 expression in genomic data and primary PDAC specimens, TGFβ suppressed PD-L1 expression in PDAC cells *in vitro* in a SMAD4-dependent manner.

To explain this discrepancy, we further explored differences between the PDAC immune microenvironment associated with SMAD4-loss. Consistent with our *in vitro* results, SMAD4-expressing tumors had a high frequency of IFNγ-producing T-cells, closely related to PD-L1 expression. As IFNγ is a potent inducer of PD-L1 expression (27), we hypothesized that the increased expression of IFNγ in SMAD4-intact tumors might overcome the suppressive effects of TGFβ/SMAD signals on PD-L1 expression in epithelial cells. Accordingly, a low dose of recombinant IFNγ strongly upregulated PD-L1 in tumor cells, irrespective of the presence of TGFβ or SMAD4. These results suggest that, though SMAD4 is a direct repressor of PD-L1 in tumor cells, by enhancing the recruitment of T-cells and raising local IFNγ levels, SMAD4 functions as an indirect inducer of PD-L1.

Given these observations, it is reasonable to hypothesize that patients with intact SMAD4 expression may be more likely to derive clinical benefit from ICI-based therapy than those with loss of SMAD4. However, it is important to note that though half of patients have SMAD4-intact disease, PDAC tumors have shown universally poor response rates to ICIs in clinical trials (8). For example, the CTLA-4 inhibitor Ipilimumab failed to produce any objective responses in PDAC (35), with similar results observed using the anti-PD-L1 antibody MPDL3280A or the anti-PD-1 antibody Pembrolizumab (36, 37). Hence, given the results of these and similar trials, it is highly unlikely that SMAD4 status will serve as a clinically useful, independent predictor of therapeutic responses to single-agent ICIs. However, select strategies combining ICIs with other treatment modalities (e.g., chemotherapy, radiation, and targeted therapy) are beginning to show promise in clinical trials (8). Hence, SMAD4 may be more informative as a predictive biomarker for such approaches, particularly in light of preclinical data suggesting that loss of SMAD4 is associated with poor responses to radiation (38) and chemotherapy (39). Additionally, *TGFBR2* is mutated in 4-7% of PDAC tumors (40, 41). Given the more modest results observed in the TGFBR2-deficient MIA PaCa-2 cell line and clinical association between *TGFBR2* mRNA and that of several T-cell surrogates, our study suggests that PDAC tumors with complete or partial loss of TGFBR2 may also have a poorly immunogenic phenotype. Hence, *TGFBR2* status should also be evaluated as a potential biomarker for responses to immunotherapy in PDAC, an associated that is now supported in lung cancer patients (42).

Beyond these more translational implications, our results also serve as an important reminder of the limitations when using mice and *in vitro* systems to study PDAC immunology. Contrasting human PDAC tumors that have a highly variable T-cell infiltrate, T-cells are largely excluded from the TME in the most widely used mouse models of PDAC, which have a myeloid-dominant TME (43, 44). This is seemingly unaffected by genetic or pharmacologic inhibition of TGFβ/SMAD signaling, as mouse models of PDAC with either genetic or pharmacologic ablation fail to demonstrate a significant increase in T-cell infiltration and display elevated levels of PD-L1 (19, 20). Hence, in the absence of IFNγ-producing T-cells, the disruption of TGFβ/SMAD signals appears to enhance PD-L1 expression similar to what was observed in cell culture. Therefore, this study affirms the need to both refine mouse models of PDAC and to incorporate complementary model systems when studying immuno-oncology such as *ex vivo* slice cultures, patient-derived xenografts in partially humanized mice, and large animal models of PDAC (45–49).

Nevertheless, our results suggest that tumors with loss of SMAD4 may comprise a unique, poorly immunogenic subtype of PDAC. Accordingly, SMAD4 status may be a clinically useful biomarker for clinical responses to ICI-based immunotherapy regimens, particularly when combined with additional predictors of therapeutic responsiveness. While this would certainly pertain to dMMR/MSI-H status, recent evidence suggests that additional genomic alterations may also contribute to the immune landscape in PDAC. Such examples include *TP53*, as it has recently been demonstrated that loss of P53 in tumor cells enhances the intratumoral accumulation of recruitment and instruction of suppressive myeloid cells, which oppose anti-cancer T-cell responses (50). Similarly, loss of the tumor suppressor gene *PTEN* has been suggested to enhance immune evasion in murine PDAC, increasing the presence of both inflammatory myeloid cells as well as immunosuppressive regulatory T-cells (Tregs) (51). Further, patients with *CDKN2A* mutations tend to have poor T- and B-cell infiltration, an increase in Tregs, and poor overall survival (52). Hence, as immunotherapy continues to advance in PDAC, SMAD4 status may warrant consideration as part of a molecular panel to predict therapeutic responses to ICIs, thereby maximizing the success of such treatment strategies and prioritizing the use of alternate approaches in patients unlikely to respond.

## Data Availability Statement

Publicly available datasets were analyzed in this study. This data can be found here: https://www.cbioportal.org.

## Author Contributions

Conception and design: DP, HM, and AR. Development of methodology: DP and AR. Acquisition of data: DP, SK, KT, and RK. Analysis and interpretation of data: DP, KT, RK HM, and AR. Writing, review, and/or revision of the manuscript: DP, AR, and HM. Administrative, technical, or material support: DP, AR, HM, PU, and JT. Funding Support: DP, HM, and AR. All authors read and approved of the final version.

## Funding

This work was supported by Veterans Affairs Merit Award I01BX004903 and Career Scientist Award IK6BX004855 to AR, by NIH F30CA236031, UIC Award for Graduate Research, and Sigma Xi Grant In the Aid of Research to DP, by NIH R01CA217907 and R21CA255291 and Veterans Affairs Merit Award I01BX002922 to HM, and by NIH R01CA242003 and the Joseph and Ann Matella Fund for Pancreatic Cancer Research to JT. Per the funding policy of the U.S. Department of Veterans Affairs, we are required to state that these contents do not represent the views of the U.S. Department of Veterans Affairs or the United States Government.

## Conflict of Interest

The authors declare that the research was conducted in the absence of any commercial or financial relationships that could be construed as a potential conflict of interest.

## Publisher’s Note

All claims expressed in this article are solely those of the authors and do not necessarily represent those of their affiliated organizations, or those of the publisher, the editors and the reviewers. Any product that may be evaluated in this article, or claim that may be made by its manufacturer, is not guaranteed or endorsed by the publisher.

## Abbreviations

PDAC, Pancreatic Ductal Adenocarcinoma; TGFβ, Transforming Growth Factor β; IFNγ, interferon γ; PD-L1, Programmed Death-Ligand 1; Dmmr, deficient in DNA mismatch repair, MSI-H, high microsatellite instability.
