# Supplementary material for: Loss of SMAD4 Is Associated With Poor Tumor Immunogenicity and Reduced PD-L1 Expression in Pancreatic Cancer
Source: Front Oncol. 2022 Jan 28;12:806963. doi: 10.3389/fonc.2022.806963 (PMC8832494; doi:10.3389/fonc.2022.806963)
Supplement: Supplementary file 7 [file Table_1.docx]

| **Antibody** | **Vendor** | **Clone Number** | **Product Number** |
| --- | --- | --- | --- |
|  |  |  |  |
| Anti-PD-L1 | abcam | EPR19759 | ab213524 |
| Anti-HLA,A,B,C | - | EMR8-5 | ab70328 |
| Anti-CD45 | - | EP322Y | ab40763 |
| Anti-IFNγ | - | EPR21704 | ab231036 |
| Anti-Mouse 488 | - | Not Provided | ab150117 |
| Anti-Rat 488 | - | Not Provided | ab96971 |
| Anti-pSMAD2 | CST | 138D4 | 3108S |
| Anti-E-Cadherin | - | 4A2 | 14472S |
| Anti-PD-L1 | - | E1L3N | 13684S |
| Anti-CD3 | SCBT | PC3/188A | sc-20047 |
| Anti-SMAD4 | - | B-8 | sc-7966 |
| Anti-GAPDH | - | 0411 | sc-47724 |
| Anti-CK19 | University of Iowa | TROMA-III | TROMA-III-c |
| Anti-IFNγ-PE | BioLegend | B27 | 506507 |
| Anti-CD69-APC | BioLegend | FN50 | 310910 |
| Anti-Mouse HRP | DAKO | Not Provided | K4001 |
| Anti-Rabbit HRP | - | Not Provided | K4003 |
| Anti-Rabbit 594 | Invitrogen | Not Provided | A11037 |

**Table S1. Antibodies arranged by vendor**

Each commercially available antibody used in this manuscript is presented in the above table and has been arranged by vendor with both the clone and product number displayed.
