# Supplementary material for: Loss of SMAD4 Is Associated With Poor Tumor Immunogenicity and Reduced PD-L1 Expression in Pancreatic Cancer
Source: Front Oncol. 2022 Jan 28;12:806963. doi: 10.3389/fonc.2022.806963 (PMC8832494; doi:10.3389/fonc.2022.806963)
Supplement: Supplementary file 8 [file Table_2.docx]

| **Patient** | **T-stage** | **N stage** | **Differentiation** | **Sex** | **Surgery Type** | **Neoadjuvant** | **SMAD4** | **T-Cells** | **Months OS** |
| --- | --- | --- | --- | --- | --- | --- | --- | --- | --- |
| 1 | 2 | 1 | Differentiated | Male | Whipple | None | Negative | Low | 7.7 |
| 2 | 2 | 1 | Undifferentiated | Male | Distal Pancreatectomy | None | Negative | Low | 8.4 |
| 3 | 3 | 2 | Differentiated | Male | Whipple | None | Positive | High | 8.8 |
| 4 | 3 | 1 | Undifferentiated | Male | Whipple | None | Negative | Low | 10.2 |
| 5 | 2 | 2 | Differentiated | Female | Distal Pancreatectomy | None | Positive | Low | 12.8 |
| 6 | 3 | 2 | Undifferentiated | Female | Whipple | None | Positive | High | 16.3 |
| 7 | 3 | 1 | Undifferentiated | Male | NR | None | Negative | Low | 20.1 |
| 8 | 3 | 0 | Undifferentiated | Male | Distal Pancreatectomy | None | Negative | Low | 22.7 |
| 9 | 3 | 0 | Differentiated | Female | Distal Pancreatectomy | None | Negative | Low | 23.8 |
| 10 | 2 | 0 | Differentiated | Female | Whipple | None | Positive | Low | 27.5 |
| 11 | 3 | 2 | Undifferentiated | Female | Whipple | None | Positive | High | 33.2 |
| 12 | 2 | 1 | Differentiated | Male | Whipple | None | Positive | High | 33.8 |
| 13 | 3 | 2 | Differentiated | Male | Whipple | None | Negative | Low | 34.4 |
| 14 | 2 | 1 | Undifferentiated | Female | Whipple | None | Positive | High | 34.5 |
| 15 | 3 | 1 | Differentiated | Male | Distal Pancreatectomy | None | Positive | High | 41.9 |
| 16 | 3 | 1 | Differentiated | Male | Distal Pancreatectomy | None | Negative | High | 46.7 |
| 17 | 3 | 0 | Differentiated | Male | Distal Pancreatectomy | None | Positive | High | 47.4 |
| 18 | 2 | 0 | Differentiated | Male | Whipple | None | Positive | High | 49.1 |
| 19 | 2 | 2 | NR | Male | Whipple | Gem/abraxane | Negative | Low | 3.2 |
| 20 | 3 | 1 | Differentiated | Male | Whipple | Gem | Negative | Low | 3.7 |
| 21 | 3 | 1 | Undifferentiated | Female | Whipple | Gem/abraxane | Positive | High | 4.7 |
| 22 | 2 | 0 | Differentiated | Male | Whipple | 5-FU/Gem | Negative | Low | 4.9 |
| 23 | 3 | 1 | Undifferentiated | Male | Whipple | Gem | Negative | Low | 5.9 |
| 24 | 3 | 0 | Differentiated | Female | Whipple | Gem/abraxane | Negative | Low | 10.3 |
| 25 | NR | 0 | NR | Female | Whipple | Gem/abraxane | Negative | Low | 11.2 |
| 26 | 3 | 1 | Differentiated | Female | Whipple | Gem/abraxane | Positive | High | 13 |
| 27 | 3 | 0 | Differentiated | Female | Distal Pancreatectomy | Gem | Positive | Low | 14.3 |
| 28 | 3 | 2 | Undifferentiated | Female | Distal Pancreatectomy | Gem/abraxane | Negative | High | 19 |
| 29 | 3 | 1 | Differentiated | Male | Whipple | Gem/abraxane | Positive | High | 23 |
| 30 | 2 | 0 | Differentiated | Female | Whipple | Gem/abraxane | Positive | High | 24.6 |
| 31 | 2 | 0 | Undifferentiated | Male | Distal Pancreatectomy | Gem/abraxane | Negative | Low | 26.6 |
| 32 | 2 | 1 | Differentiated | Male | Whipple | Gem/abraxane | Positive | High | 35.8 |
| 33 | 1 | 0 | NR | Male | Whipple | Gem/abraxane | Positive | Low | 36.1 |
| 34 | 1 | 0 | Undifferentiated | Female | Whipple | Gem/abraxane | Positive | High | 37.4 |
| 35 | 2 | 0 | Differentiated | Female | Whipple | Gem/abraxane | Negative | High | 39.1 |
| 36 | 3 | 0 | Undifferentiated | Male | Whipple | Gem/abraxane | Positive | High | 63 |

**Table S2. Clinical characteristics of PDAC patient cohort**

Not Recorded (NR); Gemcitabine (Gem); Overall Survival (OS).
