# Supplementary material for: Loss of SMAD4 Is Associated With Poor Tumor Immunogenicity and Reduced PD-L1 Expression in Pancreatic Cancer
Source: Front Oncol. 2022 Jan 28;12:806963. doi: 10.3389/fonc.2022.806963 (PMC8832494; doi:10.3389/fonc.2022.806963)
Supplement: Supplementary file 9 [file Table_3.docx]

| **Correlated Gene** | **Cytoband** | **Spearman's Correlation** | **p-Value** | **q-Value** |
| --- | --- | --- | --- | --- |
| CCL2 | 17q12 | 0.265 | 1.075e-3 | 2.955e-3 |
| CCL4 | 17q12 | 0.331 | 3.834e-5 | 1.567e-4 |
| CCL5 | 17q12 | 0.336 | 2.817e-5 | 1.189e-4 |
| CCL8 | 17q12 | 0.199 | 0.0151 | 0.0301 |
| CCL11 | 17q12 | 0.252 | 1.960e-3 | 5.012e-3 |
| CCL14 | 17q12 | 0.535 | 2.15e-12 | 7.92e-11 |
| CCL16 | 17q12 | 0.406 | 2.73e-7 | 2.007e-6 |
| CCL17 | 16q21 | 0.217 | 7.869e-3 | 0.0170 |
| CCL19 | 9p13.3 | 0.395 | 6.10e-7 | 4.061e-6 |
| CCL21 | 9p13.3 | 0.453 | 6.82e-9 | 8.09e-8 |
| CCL22 | 16q21 | 0.275 | 6.946e-4 | 2.002e-3 |
| CCL23 | 17q12 | 0.298 | 2.181e-4 | 7.217e-4 |
| CCL25 | 19p13.2 | 0.365 | 4.793e-6 | 2.494e-5 |
| CXCL9 | 4q21.1 | 0.305 | 1.525e-4 | 5.251e-4 |
| CXCL12 | 10q11.21 | 0.561 | 9.54e-14 | 6.04e-12 |
| CXCL13 | 4q21.1 | 0.357 | 7.743e-6 | 3.796e-5 |
| CXCL16 | 17p13.2 | -0.197 | 0.0161 | 0.0317 |
| CXCL17 | 19q13.2 | -0.211 | 9.754e-3 | 0.0206 |
| IL1A | 2q14.1 | -0.222 | 6.483e-3 | 0.0143 |
| IL2 | 4q27 | 0.308 | 1.305e-4 | 4.579e-4 |
| IL6 | 7p15.3 | 0.302 | 1.800e-4 | 6.086e-4 |
| IL10 | 1q32.1 | 0.323 | 5.805e-5 | 2.242e-4 |
| IL12B | 5q33.3 | 0.302 | 1.837e-4 | 6.196e-4 |
| IL12A | 3q25.33 | 0.295 | 2.618e-4 | 8.480e-4 |
| IL13 | 5q31.1 | 0.235 | 3.967e-3 | 9.344e-3 |
| IL15 | 4q31.21 | 0.193 | 0.0183 | 0.0355 |
| IL16 | 15q25.1 | 0.438 | 2.38e-8 | 2.41e-7 |
| IL17F | 6p12.2 | 0.241 | 3.051e-3 | 7.396e-3 |
| IL17D | 13q12.11 | 0.200 | 0.0145 | 0.0290 |
| IL17C | 16q24.2 | -0.183 | 0.0259 | 0.0481 |
| IL18 | 11q23.1 | -0.231 | 4.555e-3 | 0.0105 |
| IL24 | 1q32.1 | 0.355 | 9.056e-6 | 4.370e-5 |
| IL26 | 12q15 | 0.238 | 3.519e-3 | 8.403e-3 |
| IL33 | 9p24.1 | 0.475 | 9.69e-10 | 1.48e-8 |
| IL34 | 16q22.1 | 0.214 | 8.731e-3 | 0.0187 |
| IL36G | 2q14.1 | -0.275 | 6.746e-4 | 1.951e-3 |
| IL36B | 2q14.1 | -0.264 | 1.130e-3 | 3.090e-3 |

**Table S3. SMAD4 mRNA significantly associates with that of several CCL, CXCL, and IL family cytokines and chemokines in the TCGA cohort**

Using the TCGA genomic database of pancreatic cancer patients (N=186), the 149 fully sequenced tumors were evaluated for the relationship between *SMAD4* mRNA expression and that of several CCL, CXCL, and IL family cytokines and chemokines. S = Spearman correlation coefficient.
